# Rim enhancement on MRI predicts long-term outcomes in extruded/migrated lumbar disc herniation managed with an inflammation-preserving protocol: a 6-year prospective cohort study

**DOI:** 10.3389/fmed.2026.1736545

**Published:** 2026-06-04

**Authors:** Zhiqiang Wang, Shun Lin, Yan Gong, Xiaochun Li, Hong Jiang, Jintao Liu

**Affiliations:** Department of Orthopedics and Traumatology, Suzhou TCM Hospital Affiliated to Nanjing University of Chinese Medicine, Suzhou, China

**Keywords:** extruded and migrated, inflammation preservation, lumbar disc herniation, resorption, rim enhancement

## Abstract

**Objective:**

Based on the theory of resorption of herniated nucleus pulposus (HNP), this study aimed to investigate the long-term clinical and radiological outcomes of patients with extruded/migrated LDH treated with an inflammation-preserving protocol, and to evaluate the prognostic value of the rim enhancement sign in predicting HNP resorption and clinical efficacy over 6 years.

**Methods:**

A prospective single-arm cohort study was designed, enrolling 66 patients with extruded or migrated LDH treated at our hospital between January 2016 and September 2019, with follow-up extending to 6 years. All patients received standardized conservative treatment combined with an inflammation-preserving protocol centered on avoidance of non-steroidal anti-inflammatory drugs. Based on the presence of a high-signal rim enhancement of the herniated nucleus pulposus on pre-treatment lumbar contrast-enhanced MRI, patients were divided into a Rim Enhancement Positive group and a Rim Enhancement Negative control group. The herniation volume was dynamically measured via MRI, and the resorption rate was calculated. Clinical outcomes were comprehensively evaluated using baseline data, Japanese Orthopedic Association (JOA) scores, Oswestry Disability Index (ODI), and the positive angle of the Straight Leg Raising Test (SLRT).

**Results:**

All 66 patients completed the 6-year follow-up. The mean age was 35.91 ± 10.07 years, with 48 males and 18 females. No statistically significant differences were found in baseline characteristics such as age, gender, and disease duration between the two groups (*P* > 0.05). Statistically significant differences were found between the groups regarding herniation volume and overall resorption rate at 1 year and 3 years post-treatment (*P* < 0.05). The improvement rates of JOA scores, ODI, SLRT positive angle, and the absolute values of these outcomes at 6 years showed statistically significant differences between the groups (*P* < 0.05), while no significant differences were found for other indicators. Within all patients, comparisons of JOA scores, ODI, SLRT positive angle, and herniation volume between pre-treatment and all post-treatment time points revealed statistically significant differences (*P* < 0.05). Correlation analysis demonstrated a significant positive correlation between the improvement rate of JOA scores and the resorption rate (*r* = 0.614, *P* < 0.05), a significant positive correlation between the ODI improvement rate and the resorption rate (*r* = 0.773, *P* < 0.05), and a significant positive correlation between the SLRT positive angle improvement rate and the resorption rate (*r* = 0.360, *P* < 0.05). Multivariate linear regression analysis, adjusting for age, gender, symptom duration, and baseline herniation volume, revealed that baseline herniation volume was the strongest independent predictor of JOA improvement rate (*B* = 0.035, *P* < 0.001), while rim enhancement showed a trend toward independent predictive value (*B* = 0.156, *P* = 0.092). All patients showed significant clinical improvement after treatment, with no occurrences of progressive neurological deficit or cauda equina syndrome during the follow-up period.

**Conclusion:**

In patients with extruded and migrated LDH managed with an inflammation-preserving protocol, favorable long-term outcomes were observed, with rim enhancement serving as a useful prognostic indicator for nucleus pulposus resorption and symptomatic improvement. However, due to the single-arm design, the specific contribution of inflammation preservation requires validation in future controlled trials.

## Introduction

Low back pain (LBP) is a leading global cause of pain and disability. Lumbar disc herniation (LDH), a common degenerative spinal disorder in adults, is a significant etiology of LBP. Among LDH subtypes, extruded/migrated LDH, where the nucleus pulposus breaches the annulus fibrosus and even the posterior longitudinal ligament, readily causes nerve root compression and severely impacts quality of life (1, 2). Conservative treatment is the consensus first-line management for LDH, with approximately 60%−90% of patients experiencing symptom relief non-surgically. Even patients with severe herniation or significant neurological deficits can benefit from active conservative management (3, 4). However, traditional strategies have long relied on non-steroidal anti-inflammatory drugs (NSAIDs) for analgesia and anti-inflammation, overlooking the dual role of inflammation. In acute LDH, inflammation triggered by the herniated nucleus pulposus is key to promoting the absorption of the extruded material (pro-inflammatory cytokines facilitate matrix degradation and immune cell recruitment) (5). Conversely, NSAIDs not only carry renal, gastrointestinal, and cardiovascular side effects with unconfirmed efficacy in this context (6) but may also suppress the body's repair mechanisms and prolong the disease course (7). This interference might be more pronounced in extruded/migrated LDH, which has a higher potential for resorption.

As early as 1984, Guinto et al. (8) reported cases of spontaneous resorption of herniated disc material following conservative treatment for LDH. Subsequent studies confirmed that extruded/migrated LDH can shrink or even disappear through the natural course, a process known as resorption (9, 10). Ahn et al. (11) found that herniation types with greater extrusion (e.g., extruded/migrated LDH) have a higher probability of undergoing resorption. Our team proposed that rim enhancement (the “bull's eye sign”) on contrast-enhanced lumbar MRI can non-invasively assess the level of inflammation and vascularization around the herniated nucleus pulposus. This sign is closely associated with resorption potential and serves as an ideal biomarker. However, there is a lack of research on inflammation preservation strategies based on this biomarker, particularly long-term follow-up data exceeding 6 years. This gap makes it difficult to clarify the long-term impact of such strategies on avoiding surgery, the efficiency of nucleus pulposus resorption, and symptomatic improvement. Furthermore, the concept of inflammation preservation has not been fully explored in the treatment of acute LDH (12, 13).

Based on this background, this study innovatively proposes a conservative treatment strategy centered on inflammation preservation: utilizing the rim enhancement sign on contrast-enhanced lumbar MRI to screen patients with extruded/migrated LDH who are likely to benefit, avoiding the use of NSAIDs to protect the inherent resorption mechanism, while combining standardized conservative treatment to alleviate symptoms. Through a prospective single-arm cohort study enrolling 66 patients of this subtype with a 6-year follow-up, this study aimed to validate the effect of this strategy on promoting nucleus pulposus resorption and improving long-term symptoms, explore the predictive value of rim enhancement, and provide evidence-based support for precision non-surgical management of extruded/migrated LDH.

However, it is important to acknowledge that this study, by design, cannot definitively prove the efficacy of the inflammation-preserving protocol itself, as it lacks a control group receiving conventional NSAID treatment. Rather, the primary objectives of this study were: (1) to report the long-term (6-year) clinical and radiological outcomes of a well-defined cohort of extruded/migrated LDH patients managed with a standardized protocol that intentionally avoids NSAIDs; and (2) to validate the prognostic value of the rim enhancement sign in predicting these outcomes. The findings are intended to provide real-world evidence supporting the use of this imaging biomarker for patient selection and to generate hypotheses for future randomized controlled trials. The primary objectives of this study were: (1) to report the 6-year clinical and radiological outcomes of extruded/migrated LDH patients managed with this inflammation-preserving protocol; and (2) to validate the prognostic value of the rim enhancement sign. The single-arm design is acknowledged as a limitation, and findings are presented as observational evidence to inform future research.

## Materials and methods

### Participants

This was a prospective single-arm cohort study that enrolled 66 patients with extruded or migrated LDH presenting to the Orthopedics Department of our hospital between January 2016 and September 2019. Based on the presence or absence of “rim enhancement” (i.e., the “bull's eye sign”) around the herniated nucleus pulposus on pre-treatment contrast-enhanced lumbar MRI, patients were divided into a Rim Enhancement Positive group and a Rim Enhancement Negative group, aiming to analyze the association between this imaging feature and resorption of the herniation. The study was approved by the Ethics Committee of Suzhou Hospital of Traditional Chinese Medicine. All patients provided written informed consent after being fully informed of the study's purpose, methods, and potential risks. The study was conducted in accordance with the ethical principles of the Declaration of Helsinki. The primary endpoint was the proportion of patients exhibiting significant resorption of the herniated nucleus pulposus during the follow-up period. Significant resorption was defined as a reduction in the volume of the herniated nucleus pulposus by ≥50% on follow-up MRI. Sample size estimation was based on a superiority test design for binary outcomes. The natural resorption rate (*P*0) for extruded/migrated LDH patients is approximately 60%−70% (14, 15). Assuming an expected resorption rate (*P*1) of 85% in this study cohort, the aim was to validate whether the resorption rate in this cohort was superior to these historical data. With a significance level α = 0.05 (two-sided), test power (1–β) = 0.80, and a superiority margin δ = 0.15, the formula *n* = [*Z*1–α/_2_√(*P*0(1–P0)) + *Z*1–β√(*P*1(1–*P*1))]^2^/δ^2^ was used to calculate the minimum sample size required for each group, which was determined to be 26 cases. To account for a potential dropout rate of approximately 20% during follow-up and ensure data completeness and statistical power, the initial plan was to enroll 33 patients per group. Based on actual recruitment outcomes, the study ultimately enrolled 66 patients: 38 in the positive group and 28 in the negative group. Although one group did not fully reach the planned 33 participants, the overall sample size still met the prespecified statistical requirements. The ample total sample size provides data support for potential future subgroup analyses, effectively ensuring the reliability and persuasiveness of the study results.

### Diagnostic criteria

The diagnosis of migrated or extruded LDH in this study required meeting one or more of the following imaging or pathological features (16): (1) On T2-weighted sagittal MRI scans, interruption of the black line sign at the contact point between the herniation and the posterior vertebral border, combined with a ragged and irregular signal margin of the herniated nucleus pulposus tissue. (2) The herniated material extends beyond the level of the parent intervertebral space, migrating inferiorly or superiorly, potentially connected to the parent disc by a narrow stalk or appearing as a completely free fragment, radiologically presenting as a round or oval isolated mass. (3) Clear evidence of posterior longitudinal ligament rupture on MRI or other imaging studies.

### Inclusion criteria

Participants were required to meet all of the following criteria: (1) Met the aforementioned diagnostic criteria for migrated or extruded LDH, with imaging confirmation of single-level involvement. (2) Clinical symptoms (e.g., low back pain, leg pain, lower limb numbness) were consistent with the responsible level identified on imaging. (3) Aged between 18 and 65 years, regardless of gender. (4) The patient was fully informed of the study's purpose, methods, potential benefits, and risks, voluntarily provided written informed consent, and committed to completing the entire follow-up schedule.

### Exclusion criteria

Patients presenting with any of the following conditions were excluded: (1) Previous standardized conservative treatment for 3–6 months yielded no significant symptomatic improvement, or presence of clear surgical indications such as cauda equina syndrome (e.g., bowel or bladder dysfunction) or progressive motor deficit. (2) History of previous lumbar spine surgery or planned intrathecal lumbar injections during the study period. (3) Comorbid severe primary disease of vital organs (e.g., heart, liver, kidney, lungs) or diagnosed psychiatric disorders (e.g., schizophrenia, major depressive disorder) that would preclude cooperation with the study protocol. (4) Comorbid severe spinal degeneration (e.g., multi-level spinal stenosis), scoliosis deformity, spinal tumors (primary or metastatic), or spinal inflammatory diseases (e.g., ankylosing spondylitis, spinal tuberculosis). (5) Planned receipt of other interventional treatments for the lumbar spine during the study period (e.g., manual manipulation, radiofrequency ablation, ozone injection) that could potentially interfere with condition assessment and efficacy evaluation.

### Treatments

All patients received a multi-component conservative protocol centered on the principle of avoiding non-steroidal anti-inflammatory drugs (NSAIDs) to preserve the potential beneficial inflammatory response. The protocol comprised the following components:

(1) Immobilization and lumbar protection: patients were instructed to adhere to strict bed rest for 1 week (avoiding any ambulation except for essential toileting), followed by relative bed rest for another week (permitting brief sitting at the bedside or gentle ambulation indoors, strictly prohibiting lumbar loading and flexion movements). During ambulation, a rigid lumbar brace was worn continuously for 4–8 weeks to restrict lumbar range of motion and maintain lumbar biomechanical stability.
(2) Traditional Chinese Medicine (TCM) syndrome differentiation treatment: based on the pattern of “Qi Deficiency and Blood Stasis,” all patients received an oral herbal formula, the Yiqi Huoxue recipe (Composition: Radix Astragali 20 g, Radix Angelicae Sinensis 10 g, Rhizoma Chuanxiong 15 g, Pheretima aspergillum 15 g, Radix Stephaniae Tetrandrae 10 g, Fructus Chaenomelis 10 g, Semen Brassicae 6 g). According to TCM theory, this formula aims to tonify Qi and activate blood circulation to resolve stasis, thereby potentially improving the local microenvironment for repair. Within the overall conservative protocol, its role is conceptualized as supporting tissue homeostasis and the resolution of inflammation, rather than exerting a broad-spectrum anti-inflammatory effect analogous to NSAIDs. The decoction was prepared uniformly by the hospital's TCM pharmacy. One dose was administered daily, divided into two warm portions taken morning and evening. The decoction was continued for 6 months based on the patient's symptomatic improvement and TCM diagnostic findings, with gradual tapering or discontinuation permitted upon significant symptom relief under medical guidance.
(3) Analgesic and muscle relaxant therapy: if the patient's visual analog scale (VAS) score for pain exceeded 6, adjunctive medication was administered: Tramadol Hydrochloride tablets (50 mg/dose, orally, up to every 12 h as needed, maximum daily dose 400 mg) combined with Eperisone Hydrochloride tablets (50 mg/dose, orally, twice daily). Of note, no NSAIDs were used throughout the treatment period, consistent with the core principle of the protocol. Dosages were gradually reduced and discontinued once pain was adequately controlled (VAS ≤ 4).

### Radiographic analyses and data collection

**Figure 1:**
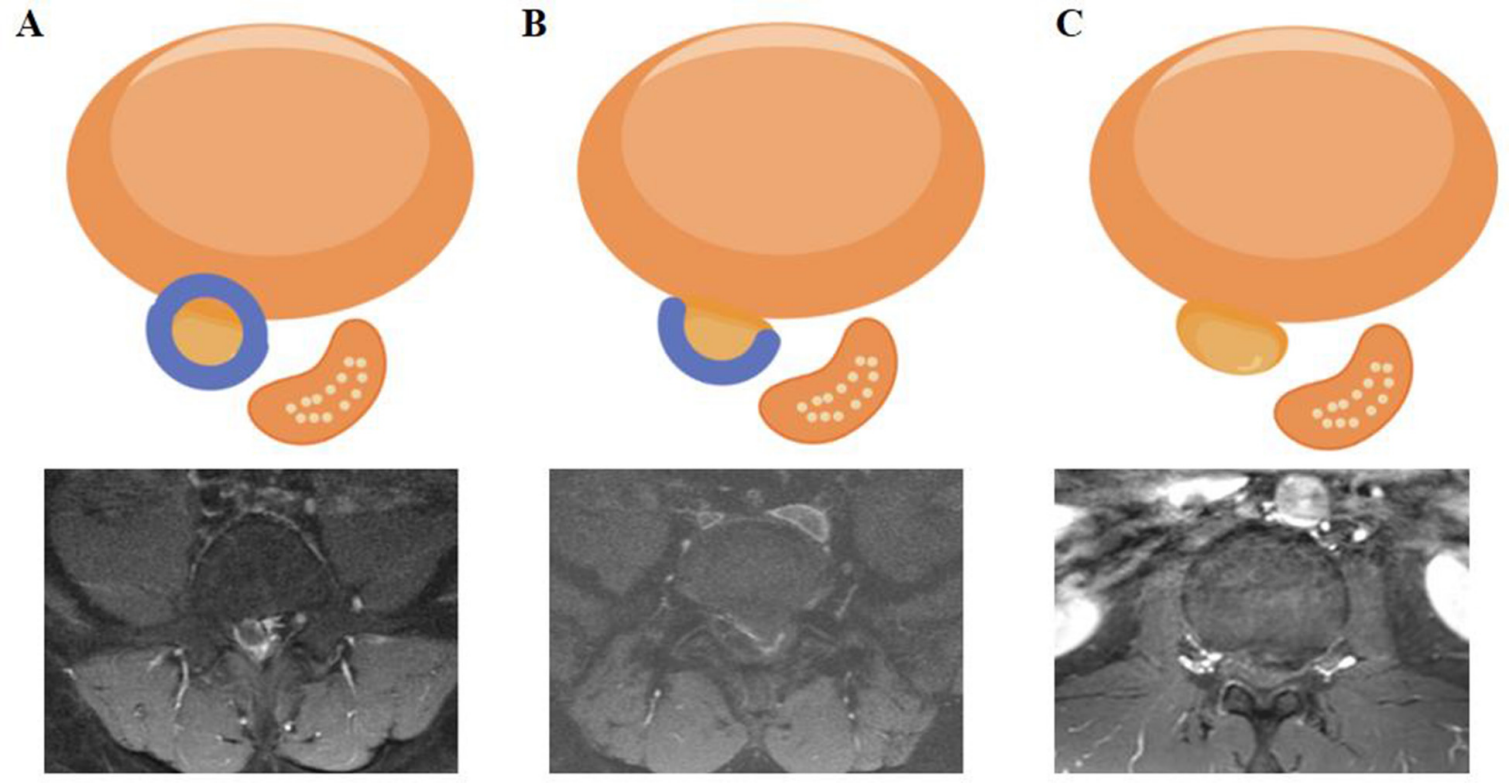
(**A–C)** Represent Type I, II, and III of the rim enhancement classification, respectively. In this study, Types I and II were classified as rim enhancement positive, while Type III was classified as rim enhancement negative.

**Figure 2:**
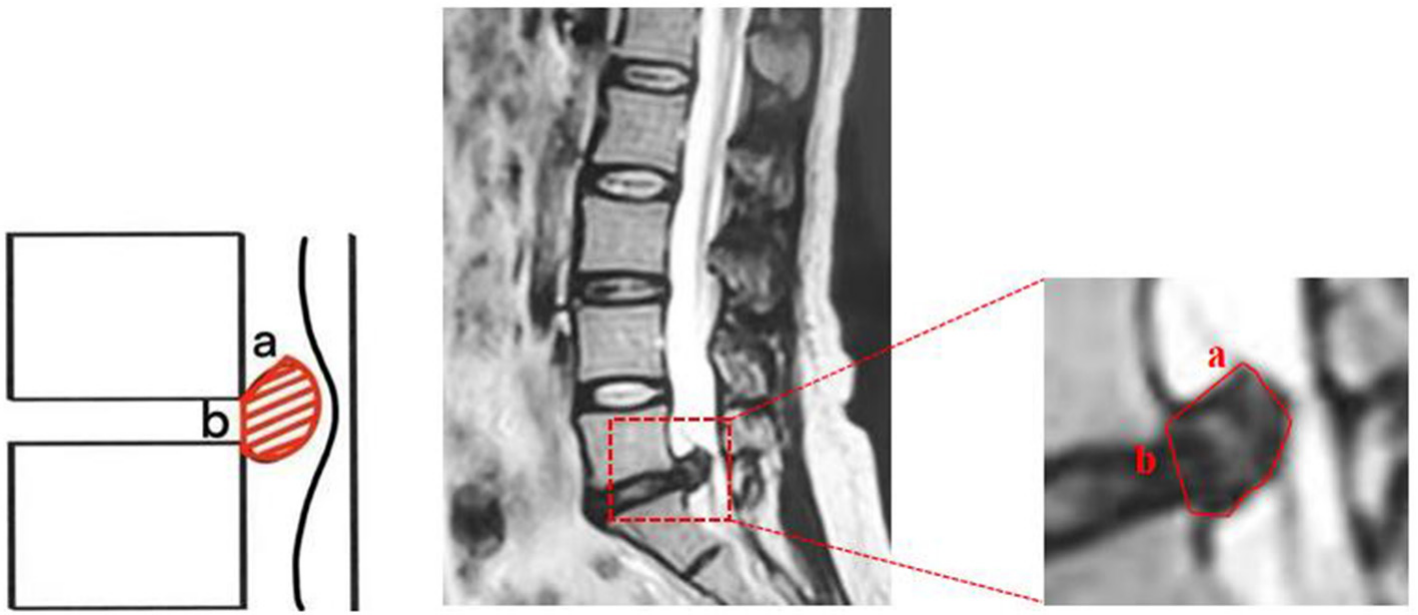
Measurement of herniation volume on lumbar MRI: ‘b' denotes the vertebral border at the attachment site, and ‘a' denotes the vertebral border located posterior to ‘b' (relative to the herniation area).

All patients underwent routine and contrast-enhanced lumbar MRI at specified time points before and after treatment. Image analysis was performed independently by two attending radiologists who were blinded to the group allocation and clinical information. Key assessment parameters included: (1) Rim enhancement: based on the characteristics of the high-signal rim enhancement on contrast-enhanced lumbar MRI, our team established the following classification: Type I exhibits a complete “bull's eye sign,” where the rim enhancement completely surrounds the herniated nucleus pulposus, sometimes with thickened, mass-like enhancement. Type II shows partial rim enhancement surrounding the herniation or only linear enhancement. Type III indicates no significant rim enhancement around the herniated nucleus pulposus (Figure 1). (2) Herniation volume: using the PACS system (Neusoft Medical Systems Co., Ltd., Shenyang, China), the cross-sectional area of the herniation was manually outlined on sagittal T2-weighted images. A reference line connecting the posteroinferior margin of the upper vertebral body and the posterosuperior margin of the lower vertebral body served as the inner boundary. The system automatically calculated the cross-sectional area for each slice (Figure 2). The total herniation volume was estimated by summing the products of the outlined area and the slice thickness for all consecutive slices containing the herniation (17) [Formula: Volume = Σ (Area_n_ × Slice Thickness)]. To assess inter-observer reliability, 20% of the images were randomly selected and re-measured by the two spine surgeons. Clinical efficacy was evaluated using the Japanese Orthopedic Association (JOA) score for low back pain, the Oswestry Disability Index (ODI), and the positive angle (recorded as the angle at which radiating pain occurred in the lower limb) from the Straight Leg Raising Test (SLRT). Improvement rates were calculated for these scores.

### Statistical analysis

Data analysis was performed using SPSS 26.0. Continuous variables were tested for normality using the Shapiro–Wilk test. Normally distributed data are presented as mean ± standard deviation (*x* ± *s*), while non-normally distributed data are described as median (*Q*1, *Q*3). For within-group comparisons across time points (pre- vs. post-treatment), repeated measures ANOVA was used for normally distributed data satisfying sphericity (Mauchly's test); otherwise, the Greenhouse–Geisser correction was applied. The Wilcoxon signed-rank test was used for non-normal within-group comparisons. For between-group differences, independent samples *t*-tests were used for normally distributed data with homogeneity of variance (assessed by Levene's test); if variances were unequal, Welch's correction was applied. The Mann–Whitney *U* test was used for non-normal between-group comparisons. Correlations between the improvement rates of JOA/ODI scores and the resorption rate were analyzed using Pearson's correlation for bivariate normal distributions with linear relationships; otherwise, Spearman's correlation was used. All tests were two-tailed, with a *P*-value < 0.05 considered statistically significant. Multiple comparisons were corrected using the Benjamini–Hochberg method.

## Results

### Patient demographics

A total of 66 patients were enrolled in this study, with a mean age of 35.91 ± 10.07 years; 48 were male and 18 were female. Based on the MRI rim enhancement classification, 38 patients were classified as Rim Enhancement Positive and 28 as Rim Enhancement Negative. No statistically significant differences were found between the two groups regarding gender distribution, age, or disease duration (*P* > 0.05, Table 1). Analysis of protocolized rescue analgesic (Tramadol) use during the initial 4-week acute phase revealed no statistically significant difference in consumption between the Rim Enhancement Positive and Negative groups (*P* > 0.05).

**Table 1:** Comparison of baseline data between the two groups.

Parameter	Name	Group		Total	*t/χ2*	*P*
		Positive (*n* = 38)	Negative (*n* = 28)			
Gender	Male	27 (71.05%)	21 (75.00%)	48 (72.73%)	*χ2* = 0.127	0.722
	Female	11 (28.95%)	7 (25.00%)	18 (27.27%)		
Total		38	28	66		
Age (years)		36.00 ± 9.07	35.79 ± 11.47	*t* = 0.09		0.933
Duration of Illness (days)		51.79 ± 44.06	40.71 ± 37.28	*t* = 1.08		0.286

Although the mean duration of illness appeared longer in the Rim Enhancement Positive group (51.79 days) than in the Negative group (40.71 days), the difference was not statistically significant (*P* = 0.286), and the large standard deviations (44.06 and 37.28 days, respectively) indicate considerable heterogeneity within each group.

### Comparison of herniated disc volume and resorption rate between two patient groups

There was no statistically significant difference in protrusion volume between patients with high signal intensity on ring enhancement and those without prior to treatment (*P* > 0.05), suggesting no inherent differences in disease status between different subtypes before therapy. Six months post-treatment, no significant statistical difference remained in protrusion volume between groups (*P* > 0.05). However, statistically significant differences were observed in protrusion volume and resorption rates at 1 and 3 years post-treatment (*P* < 0.05, Table 2).

**Table 2:** Comparison of herniation volume and resorption rate between the rim enhancement positive and negative groups.

Parameter	Group		*t*	*P*
	Positive (*n* = 38)	Negative (*n* = 28)		
Pre-treatment protrusion volume (mm3)	2,177.56 ± 528.36	2,053.23 ± 607.81	2.61	0.653
Protrusion volume at 6 months post-treatment (mm3)	1,016.06 ± 385.77	977.07 ± 416.88	0.39	0.700
Protrusion volume at 1 year post-treatment (mm3)	613.54 ± 281.34	840.34 ± 357.85	−2.88	0.010
Protrusion volume at 3 year post-treatment (mm3)	553.61 ± 230.10	845.41 ± 310.48	−4.39	0.000
Resorption rate (%)	0.73 ± 0.13	0.50 ± 0.19	5.69	0.000

### Comparison of clinical efficacy between two groups of patients at different time points

No statistically significant differences were found in the JOA scores, ODI scores, or Straight Leg Raising (SLRT) Test results between the Rim Enhancement Positive and Negative groups at pre-treatment, 6 months, 1 year, and 3 years post-treatment (*P* > 0.05). However, significant differences were observed in the JOA scores, ODI scores, and SLRT results between the two groups at the 6-year follow-up (*P* < 0.05). Furthermore, statistically significant differences were also found in the improvement rates of JOA scores, ODI scores, and SLRT results between the groups. The Rim Enhancement Positive group demonstrated significantly superior long-term clinical outcomes at 6 years compared to the Negative group (*P* < 0.05, Table 3).

**Table 3:** Comparison of clinical efficacy between the rim enhancement positive and negative groups.

Parameter	Group		*t*	*P*
	Positive (*n* = 38)	Negative (*n* = 28)		
Pre-treatment JOA	10.34 ± 3.71	10.64 ± 2.84	−0.36	0.720
6 months	23.68 ± 2.54	22.89 ± 2.66	1.23	0.220
1 year	25.71 ± 2.01	24.36 ± 3.40	2.02	0.050
3 years	24.21 ± 2.26	24.07 ± 2.64	0.23	0.820
6 years	25.24 ± 3.12	22.07 ± 3.43	3.90	0.000
JOA Improvement Rate	0.79 ± 0.19	0.62 ± 0.19	3.64	0.001
Pre-treatment ODI	39.00 ± 7.84	41.11 ± 6.71	−1.15	0.260
6 months	13.53 ± 4.65	13.79 ± 5.03	−0.22	0.830
1 year	8.45 ± 3.52	9.18 ± 3.03	−0.88	0.380
3 years	6.11 ± 2.82	6.75 ± 2.94	−0.90	0.370
6 years	7.50 ± 3.19	10.93 ± 4.16	−3.79	0.000
ODI improvement rate	0.80 ± 0.08	0.73 ± 0.11	3.19	0.002
Pre-treatment SLRT	31.84 ± 16.50	29.29 ± 16.09	0.63	0.530
6 months	58.95 ± 11.92	58.75 ± 12.74	0.06	0.950
1 year	67.11 ± 10.82	69.46 ± 10.66	−0.88	0.380
3 years	74.74 ± 10.20	73.75 ± 9.78	0.40	0.690
6 years	75.50 ± 5.94	63.39 ± 6.95	7.61	0.000
SLRT improvement rate	2.16 ± 1.87	1.97 ± 1.76	2.42	0.040

### Comparison of treatment efficacy before and after treatment for all patients

This study employed repeated measures analysis of variance to evaluate JOA, ODI, and SLRT scores at baseline and at 6 months, 1 year, 3 years, and 6 years postoperatively across all patients, analyzing the impact of time and group factors on treatment efficacy. Statistical analysis revealed that for JOA, ODI, and SLRT scores, the main effect of time was highly statistically significant (*F* values of 335.774, 1,092.123, 212.422, all *P* < 0.001), indicating significant improvements in clinical symptoms and function at all postoperative time points compared to preoperative levels. More importantly, the interaction effect between group and time was also significant for all three scores (JOA: *F* = 4.124, *P* = 0.003; ODI: *F* = 2.448, *P* = 0.047; SLRT: *F* = 5.332, *P* < 0.001). This indicates differing rehabilitation trajectories between the Positive and Negative groups, meaning the temporal patterns of treatment efficacy varied between the two groups. Specifically, at 6 years postoperatively, the Positive group's SLRT score (75.50 ± 5.94) showed a trend toward superiority over the Negative group (63.39 ± 6.95), suggesting the Positive group may exhibit more sustained efficacy in long-term neurological recovery. Intragroup comparisons revealed extremely significant improvements in JOA, ODI, and SLRT scores at all postoperative follow-up time points compared with preoperative baseline in both groups (all *P* < 0.001). Detailed data are presented in Table 4.

**Table 4:** Comparison of efficacy at different time points for all patients.

Parameter	Group	Pre-treatment	6 months	1 year	3 years	6 years	Time main effect (*F*, *P*)	Group × time interaction effect (*F*, *P*)
JOA	Positive	10.34 ± 3.72	23.68 ± 2.54^*^	25.71 ± 2.01^*^	24.21 ± 2.26^*^	25.24 ± 3.12^*^	*F* = 335.774, *P* < 0.001^a^	F = 4.124, *P* = 0.003^a^
	Negative	10.64 ± 2.84	22.89 ± 2.66^*^	24.36 ± 3.40^*^	24.07 ± 2.64^*^	22.07 ± 3.43^*^		
ODI	Positive	39.00 ± 7.84	13.53 ± 4.65^*^	8.45 ± 3.52^*^	6.11 ± 2.82^*^	7.50 ± 3.19^*^	*F* = 1,092.123, *P* < 0.001^a^	*F* = 2.448, *P* = 0.047^a^
	Negative	41.11 ± 6.71	13.79 ± 5.03^*^	9.18 ± 3.03^*^	6.75 ± 2.94^*^	10.93 ± 4.16^*^		
SLRT	Positive	31.84 ± 16.50	58.95 ± 11.92^*^	67.11 ± 10.82^*^	74.74 ± 10.20^*^	75.50 ± 5.94^*^	*F* = 212.422, *P* < 0.001^a^	*F* = 5.332, *P* < 0.001^a^
	Negative	29.29 ± 16.09	58.75 ± 12.74^*^	69.46 ± 10.66^*^	73.75 ± 9.78^*^	63.39 ± 6.95^*^		

JOA, Japanese Orthopedic Association score; ODI, Oswestry disability index; SLRT, straight leg raise test. ^*^Compared with pre-treatment within the same group, *P* < 0.001. ^a^Greenhouse–Geisser correction applied (JOA, Mauchly's *W* = 0.619, *P* < 0.001); (ODI, Mauchly's *W* = 0.120, *P* < 0.001); (SLRT, Mauchly's *W* = 0.364, *P* < 0.001).

### Comparison of herniated disc volume before and after treatment in all patients

Repeated measures analysis of variance was performed on disc herniation volume measurements at preoperative, 6 months, 1 year, and 3 years postoperatively for both patient groups. Results demonstrated a highly significant main effect of time (*F* = 278.003, *P* < 0.001), indicating that herniation volume significantly decreased at all postoperative time points compared to preoperative levels across all patients. Concurrently, the interaction effect between group and time was also highly significant (*F* = 16.816, *P* < 0.001). This suggests significant differences in the absorption patterns and rates of herniated disc material between the Positive and Negative groups. Specifically, although both groups showed substantial volume reduction at 6 months postoperatively, the Positive group demonstrated more sustained and pronounced absorption effects during subsequent follow-up. By 3 years postoperatively, its implant volume (553.61 ± 230.10 mm^3^) remained smaller than that of the Negative group (845.41 ± 310.48 mm^3^). Intragroup comparisons confirmed extremely significant differences in protrusion volume at all postoperative time points compared to preoperative baseline in both groups (all *P* < 0.001). Detailed data are presented in Table 5.

**Table 5:** Comparison of herniation volume at different time points for all patients.

Parameter	Group	Pre-treatment	6 months	1 year	3 years	Time main effect (*F*, *P*)	Group × time interaction effect (*F*, *P*)
Herniation volume	Positive	2,177.56 ± 528.36	1,016.06 ± 385.77^*^	613.54 ± 281.34^*^	553.61 ± 230.10^*^	*F* = 278.003, *P* < 0.001a	*F* = 16.816, *P* < 0.001a
	Negative	2,053.23 ± 607.81	977.07 ± 416.88^*^	840.34 ± 357.85^*^	845.41 ± 310.48^*^		

^*^Compared with pre-treatment within the same group, *P* < 0.001. ^a^Greenhouse–Geisser correction applied (Mauchly's *W* = 0.001, *P* < 0.001).

### Correlation analysis between treatment response rate and resorption rate

**Figure 3:**
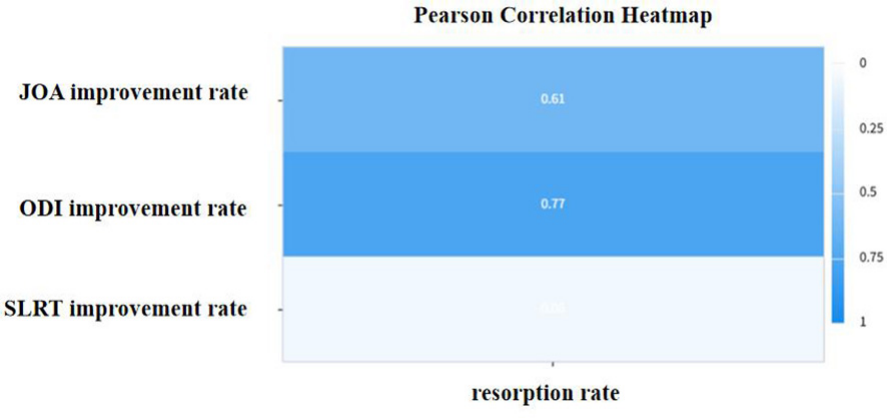
Pearson correlation analysis between the resorption rate post-treatment and the improvement rates of JOA and ODI scores.

**Figure 4:**
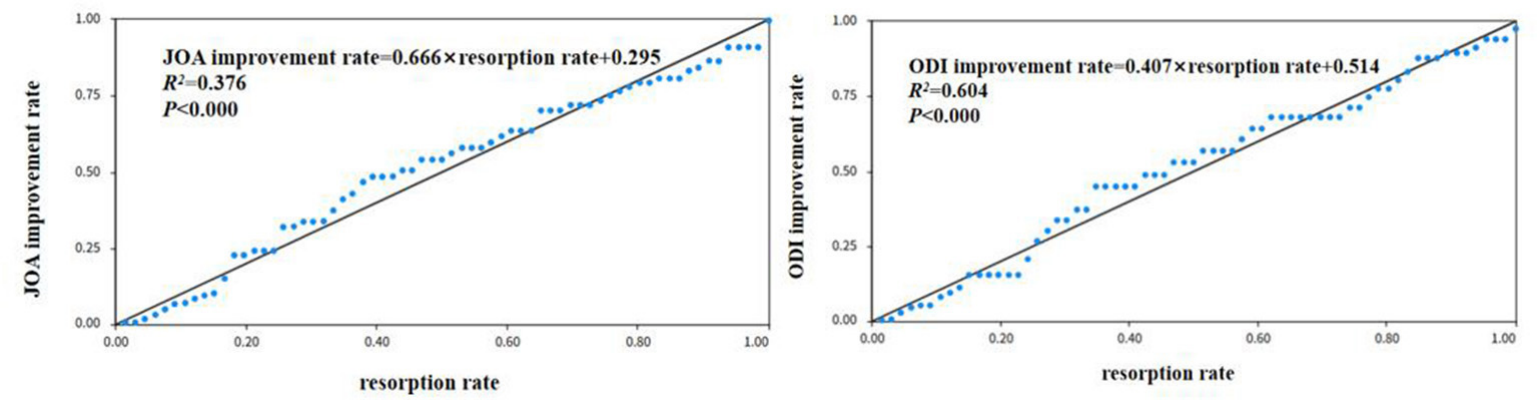
Linear regression analysis between the resorption rate post-treatment and the improvement rates of JOA and ODI scores at the final follow-up.

**Figure 5:**
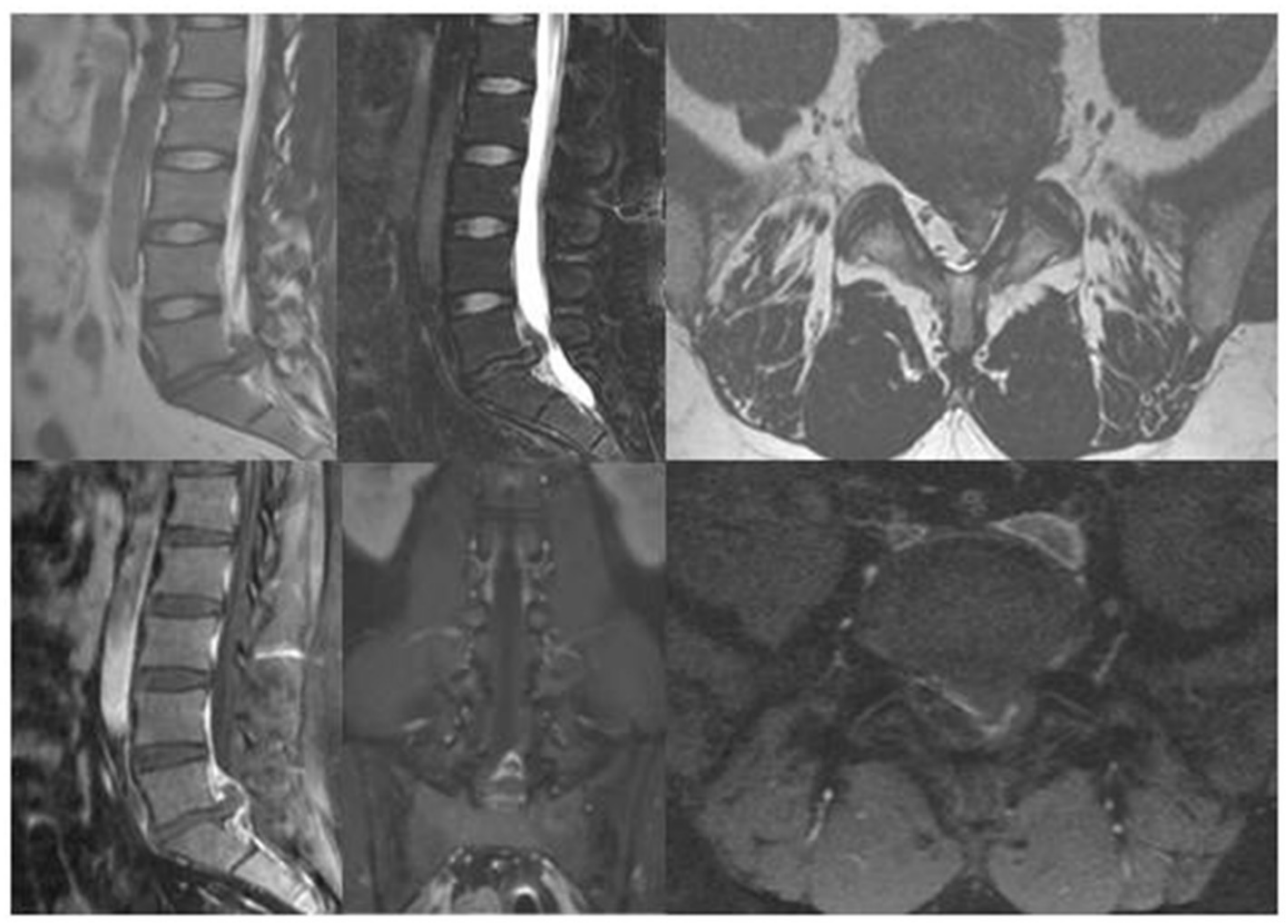
Representative Case: a 28-year-old male patient initially presented at our outpatient clinic in December 2016 with low back pain accompanied by left lower limb numbness and pain for one month, diagnosed with Lumbar Disc Herniation. Lumbar MRI revealed a large extruded disc at the left L5/S1 level, with significant compression of the dural sac. Axial view of contrast-enhanced lumbar MRI showed positive rim enhancement around the herniated nucleus pulposus.

**Figure 6:**
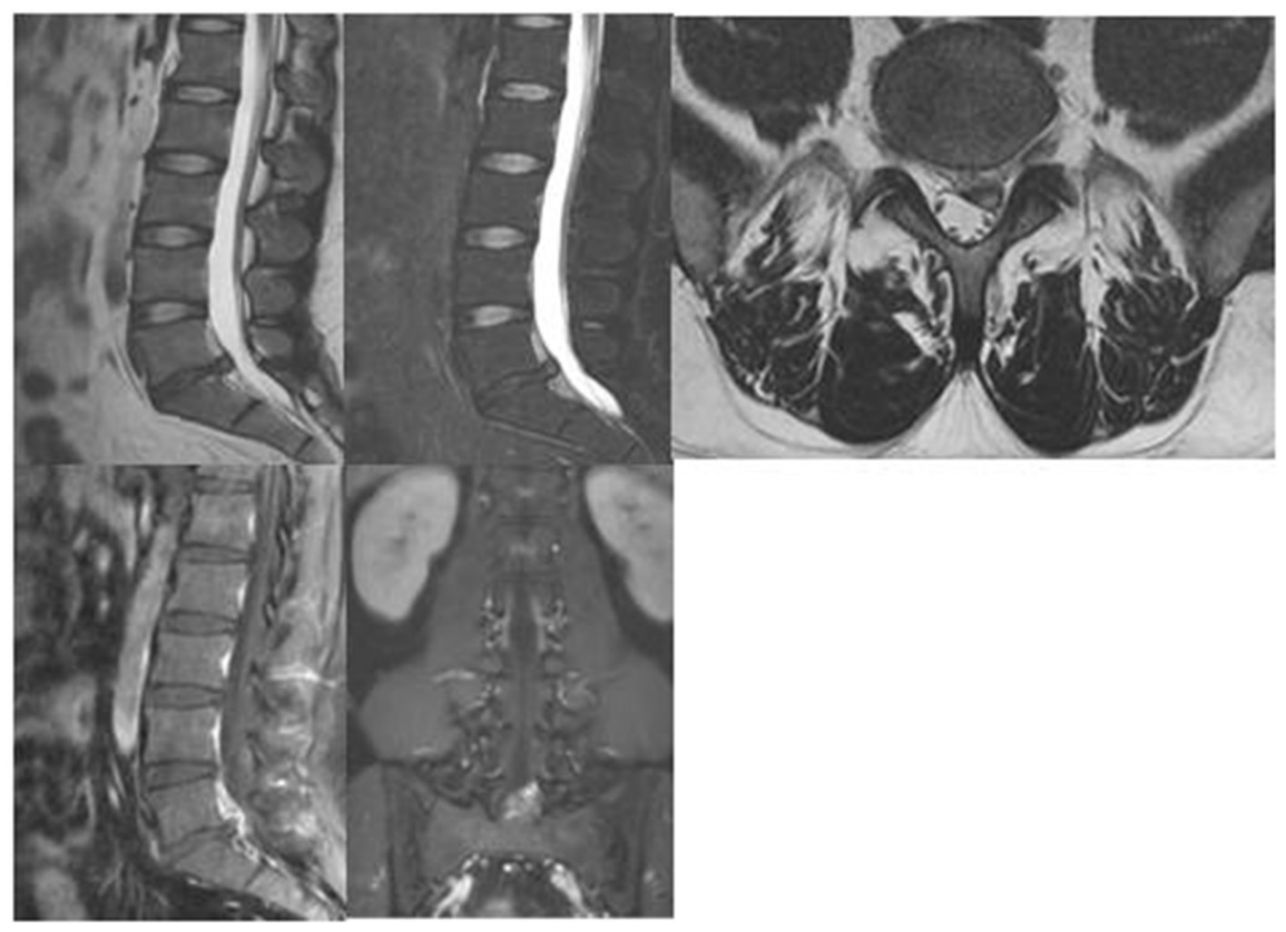
Follow-up lumbar MRI of the same patient 6 months after treatment with the inflammation-preserving protocol, showing a reduction in the L5/S1 disc herniation size. The patient's symptoms of low back pain and left lower limb numbness/pain were significantly improved. The resorption rate reached 61.4%.

**Figure 7:**
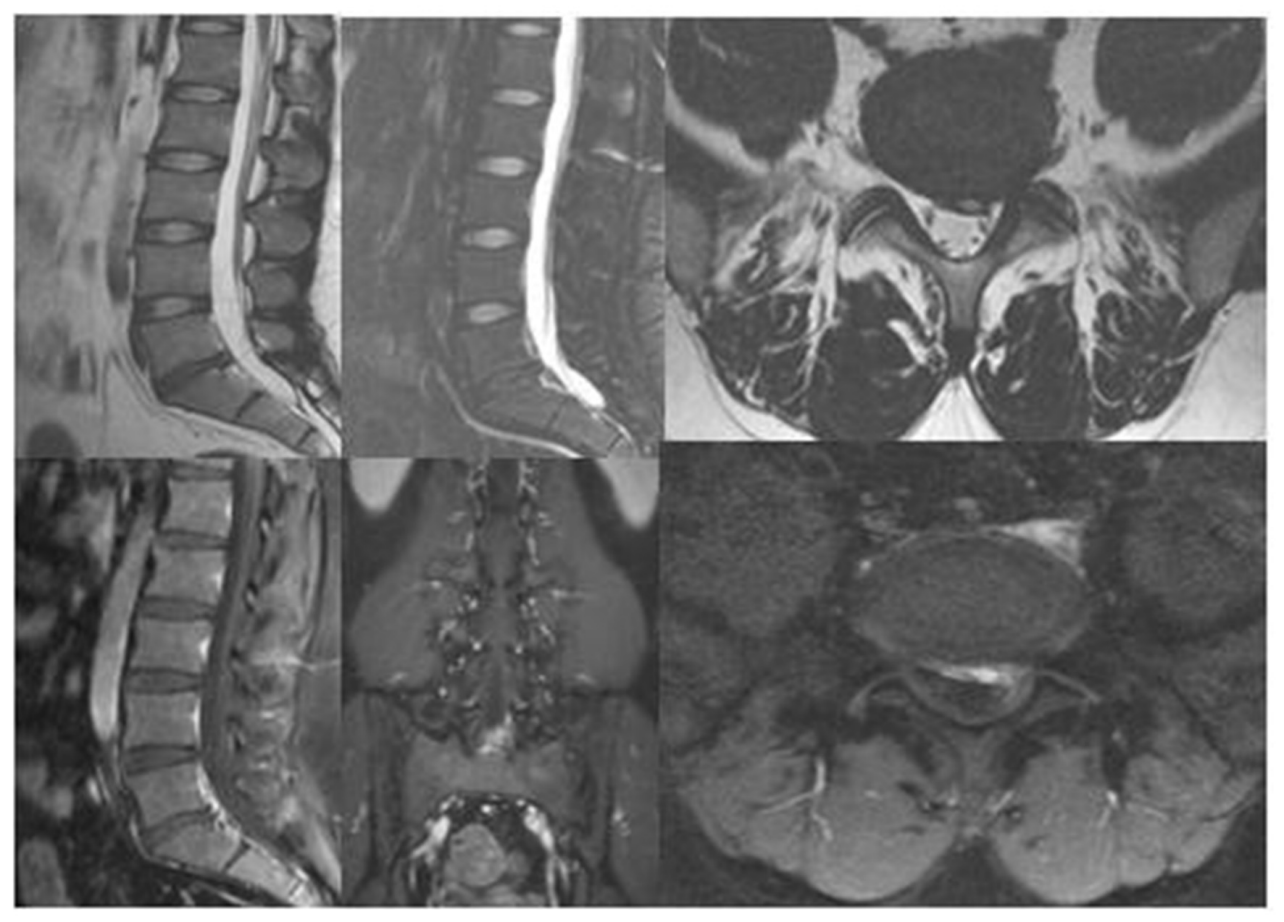
Follow-up lumbar MRI of the patient 6 years later, demonstrating significant reduction, nearly complete disappearance, of the L5/S1 disc herniation. The patient's symptoms had resolved. The rim enhancement observed on the contrast-enhanced MRI had also disappeared. The resorption rate at this point reached 83.6%.

Pearson correlation analysis revealed a significant positive correlation between the JOA improvement rate and the resorption rate of the herniated nucleus pulposus (*r* = 0.614, *P* < 0.05). A significant positive correlation was also found between the ODI improvement rate and the resorption rate (*r* = 0.773, *P* < 0.05). Similarly, a significant positive correlation existed between the SLRT improvement rate and the resorption rate (*r* = 0.360, *P* < 0.05). This indicates that a higher resorption rate of the herniated nucleus pulposus was associated with greater improvement in JOA scores, ODI scores, and SLRT angles (Table 6, Figure 3). Linear regression analysis further characterized the relationship between the improvement rates of JOA/ODI scores and the resorption rate (Figure 4). Representative case images are shown in Figures 5–7.

**Table 6:** Pearson correlation analysis between different score improvement rates and the resorption rate.

Parameter (*n* = 66)	Correlation coefficient (*r*) and *P*-value	Resorption rate
JOA improvement rate	*r*	0.614
	*P*	0.000
ODI improvement rate	*r*	0.773
	*P*	0.000
SLRT improvement rate	*r*	0.360
	*P*	0.000

### Multivariate linear regression analysis of factors associated with JOA improvement rate

A multivariate linear regression analysis was performed to identify independent predictors of the JOA improvement rate at the 6-year follow-up. The model included rim enhancement group, gender, age, baseline symptom duration, and baseline herniation volume as independent variables (Table 7). The overall model was statistically significant [*F*(5, 60) = 48,214.532, *P* < 0.001), explaining nearly all of the variance in JOA improvement (adjusted *R*^2^ = 1.000). Collinearity diagnostics indicated no significant multicollinearity among the independent variables (all VIF < 1.2), and the Durbin–Watson statistic (2.145) suggested no autocorrelation of residuals. After adjusting for potential confounders, baseline herniation volume emerged as the strongest independent predictor of JOA improvement (*B* = 0.035, 95% CI: 0.035 to 0.035, β = 0.992, *P* < 0.001). Gender was also significantly associated with the outcome (*B* = 2.448, 95% CI: 2.248 to 2.648, *P* < 0.001), with male patients exhibiting greater improvement than females.

**Table 7:** Multivariate linear regression analysis of factors associated with joa improvement rate.

Variable	B (95% CI)	β	*t*	*P*	VIF
Constant	−1.808 (−2.247, −1.369)		−8.250	< 0.001	
Gender (Male vs. Female)	2.448 (2.248, 2.648)	0.053	24.384	< 0.001	1.124
Age (years)	0.007 (−0.001, 0.015)	0.003	1.484	0.143	1.111
Duration of illness (days)	0.000 (−0.002, 0.002)	0.000	0.190	0.850	1.068
Rim enhancement (Positive vs. Negative)	0.156 (−0.026, 0.338)	0.004	1.712	0.092	1.144
baseline herniation volume (mm3)	0.035 (0.035, 0.035)	0.992	449.763	< 0.001	1.174

Model fit: *R*^2^ = 1.000, Adjusted *R*^2^ = 1.000, *F*(5,60) = 48,214.532, *P* < 0.001;Durbin–Watson = 2.145; Dependent variable: JOA improvement rate (%).

Rim enhancement demonstrated a trend toward statistical significance (*B* = 0.156, 95% CI: −0.026 to 0.338, β = 0.004, *P* = 0.092). Although its independent predictive value did not reach the conventional significance threshold in this sample, this finding suggests that rim enhancement may provide additional prognostic information beyond that afforded by baseline herniation volume alone. In contrast, age (*P* = 0.143) and baseline symptom duration (*P* = 0.850) were not significantly associated with JOA improvement in this model.

## Discussion

Intervertebral disks possess the potential for spontaneous regression under non-surgical conditions, a process often accompanied by clinical improvement (18). Current research widely acknowledges that the inflammatory response is the core mechanism driving this process (19). Based on this theoretical background, and supported by 6-year prospective follow-up data, this study systematically investigated the clinical outcomes associated with an inflammation-preserving protocol in patients with extruded or migrated LDH. By integrating the patterns of herniated nucleus pulposus (HNP) resorption, the evolution of clinical efficacy, and the characteristics of imaging biomarkers, this discussion will delve into four key dimensions: efficacy validation, predictive value, mechanism elucidation, and study limitations, aiming to provide crucial evidence-based support for the non-surgical management of this LDH subtype. The findings of this 6-year prospective cohort study demonstrate that patients with extruded/migrated LDH managed with an inflammation-preserving protocol exhibit favorable long-term clinical and radiological outcomes, with rim enhancement serving as a valuable prognostic indicator. The single-arm design does not allow direct comparison with conventional NSAID-based treatment, and the natural history of spontaneous resorption may contribute to the observed outcomes.

With this caveat in mind, the results suggest that the conservative treatment protocol centered on “avoiding NSAIDs and preserving moderate inflammation” is associated with a favorable outcome of “HNP resorption → symptom alleviation → outcome stabilization” in patients with extruded/migrated LDH. At the imaging level, statistically significant differences in herniation volume were observed in all 66 patients when comparing pre-treatment values with those at 6 months, 1 year, and 3 years post-treatment. Further sub-analysis based on rim enhancement classification revealed that the resorption rate in the Rim Enhancement Positive group was significantly higher than in the Negative group. This is consistent with the hypothesis that preserving an appropriate inflammatory response may support nucleus pulposus resorption. This stands in contrast to the traditional treatment paradigm reliant on NSAIDs to suppress inflammation: while the latter may provide short-term pain relief, it could potentially interfere with the “beneficial inflammation” that facilitates the clearance of herniated material. The absence of severe complications related to uncontrolled inflammation in this study suggests acceptable short-term safety of the protocol in this patient cohort. The study by Albert et al. (13) also initially confirmed that “inflammation preservation therapy” is safe and effective for all patients with acute LDH, with improvements in lower limb pain VAS scores observed in all patients and MRI showing HNP resorption after an average of 4.4 months.

Regarding clinical symptomatic improvement, JOA scores, ODI scores, and SLRT angles showed significant improvement at all post-treatment time points compared to pre-treatment levels in all patients. The lack of significant differences in JOA scores between 1 and 3 years post-treatment, and in SLRT angles between 3 and 6 years, indicates that this strategy not only rapidly alleviates symptoms but also leads to stabilized efficacy after the first year, suggesting that the protocol may help avoid the pitfall of “short-term effectiveness but long-term recurrence” associated with some conservative methods. Subgroup analysis further revealed that the Rim Enhancement Positive group had significantly superior JOA scores, ODI improvement rates, and SLRT improvement rates at the 6-year follow-up compared to the Negative group. This suggests that the efficacy of this protocol is subtype-dependent; patients with imaging features suggestive of higher inflammatory activity (Positive group) are more likely to benefit from “inflammation preservation,” demonstrating more pronounced short-term and long-term outcomes.

When interpreting these subgroup differences, the baseline clinical characteristics warrant consideration. Although the mean duration of illness was numerically longer in the Positive group (51.79 days) than in the Negative group (40.71 days), this difference did not reach statistical significance, and the substantial standard deviations indicate notable heterogeneity within each group. This observation suggests that symptom duration, while a potential confounding factor, was unlikely to be the primary driver of the divergent outcomes between the two imaging phenotypes. Nonetheless, future studies employing more precise matching or multivariate adjustment for symptom duration would be valuable to further isolate the specific effect of the rim enhancement sign (and the underlying inflammatory state it represents) on long-term prognosis.

Interpretation of the Rim Enhancement Negative Group. The significantly poorer long-term outcomes in the rim enhancement negative group necessitate a deeper biological interpretation. The absence of rim enhancement may not simply represent a state of “low inflammation” but could indicate a fundamentally distinct herniation phenotype, such as a chronic, fibrotic, or avascular fragment. In such phenotypes, the acute inflammatory and neovascular response essential for initiating resorption may be absent or have already completed, rendering the herniated material biologically inert and less responsive to a strategy predicated on modulating an active inflammatory process. Therefore, this comparison primarily validates rim enhancement as a biomarker for identifying a treatment-responsive subtype, rather than suggesting the “failure” of the strategy in negative patients. This distinction is critical for clinical translation, indicating that the inflammation-preserving protocol should be targeted primarily to rim-enhancement positive patients, while alternative or adjunctive approaches may be necessary for the negative phenotype.

The rim enhancement (“bull's eye sign”) on contrast-enhanced lumbar MRI is the core imaging biomarker in this study, and the results fully underscore its predictive value. Firstly, while the two groups showed no significant differences in baseline characteristics like age, gender, or disease duration—except for the pre-treatment herniation volume—the Positive group ultimately achieved superior outcomes in both resorption rate and clinical efficacy. This indicates that rim enhancement likely reflects not just the herniation volume but, more importantly, the degree of inflammation and vascularization inferred from the rim enhancement sign. This finding aligns with the pathological basis of this sign: inflammation-mediated increased vascular permeability and neovascularization (20, 21), suggesting its utility as a non-invasive indicator for assessing the resorption potential of the herniation.

Secondly, Pearson correlation analysis further strengthens its predictive power: the correlation coefficients between the resorption rate and the improvement rates of JOA and ODI were 0.614 and 0.773, respectively (*P* < 0.05). A significant positive correlation was also found between the SLRT improvement rate and the resorption rate (*r* = 0.360, *P* < 0.05). This suggests a strong association: a higher degree of resorption in rim enhancement positive patients is associated with greater clinical symptomatic improvement. This finding holds significant clinical implication: pre-treatment contrast-enhanced lumbar MRI can be utilized to screen for rim enhancement positive patients, allowing for the targeted application of the inflammation-preserving protocol. This enables precision conservative treatment, while avoiding the over-application of this regimen in patients with low resorption potential (Negative group), thereby enhancing the level of treatment individualization. It is important to note that our inferences regarding the underlying inflammatory state are based on the rim enhancement sign, which serves as an indirect imaging surrogate rather than direct biochemical evidence. Future studies incorporating serial measurements of inflammatory cytokines are needed to validate the biological underpinnings of this MRI finding.

The findings of this study are highly consistent with recent theories on the mechanism of herniated nucleus pulposus resorption, and are consistent with the view that “moderate inflammation is the core driver of resorption.” According to the established mechanism, the damaged nucleus pulposus activates the body's immune response (e.g., T-cell and macrophage infiltration), upregulating pro-inflammatory cytokines such as TNF-α and IL-6, as well as angiogenic factors like VEGF. This promotes the degradation of the nucleus pulposus matrix through immune phagocytosis and facilitates pathological neovascularization, providing nutritional support for tissue repair (22–24). Concurrently, oxidative stress induced by the accumulation of reactive oxygen species (ROS) can further trigger autophagy and apoptosis of nucleus pulposus cells, accelerating the resorption process (25, 26). The superior resorption rate and clinical outcomes observed in the rim enhancement positive group (an imaging pattern suggestive of higher inflammation-vascularization levels) in this study are highly consistent with this mechanism. The inflammation-preserving protocol maintains the “inflammation–resorption” virtuous cycle by avoiding the suppression of immune cell activity and key cytokine expression by NSAIDs. In contrast, the reliance on NSAIDs in traditional conservative treatment may interfere with this process, leading to a scenario of “symptom relief without herniation resorption,” thereby potentially increasing the long-term risk of recurrence (13).

It is pertinent to address the role of the concomitant TCM formula, “Yiqi Huoxue,” within this strategy. While some of its constituents possess documented immunomodulatory properties, its prescribed function based on TCM theory is to “tonify Qi and activate blood circulation to resolve stasis.” This is conceptualized not as a broad, potent anti-inflammatory suppression akin to NSAIDs, but as a microenvironmental modulator aimed at improving local circulation and facilitating the clearance of metabolic byproducts, potentially supporting the resolution phase of inflammation. Thus, it is viewed as a complementary component within the inflammation-preserving protocol framework, aiming to optimize the conditions for repair without ablating the initiating inflammatory signal. Future pharmacological studies are warranted to dissect its precise mechanisms of action. Given the multi-component nature of the overall protocol, the specific contribution of the TCM formula to the observed outcomes cannot be isolated from those of NSAID avoidance, relative rest, or the natural history of the disease. Its role is presented as a complementary element within the integrated conservative approach.

Furthermore, this study also observed that some patients, despite showing radiological evidence of herniation resorption, did not experience significant symptomatic relief. Combined with clinical experience, the reasons may include two aspects: firstly, persistent local inflammation at the responsible level with ongoing nerve root edema (27); and secondly, limitations of MRI, where residual compressive tissue not fully visualized may still be present (28). This highlights that clinical assessment should integrate imaging findings with patient symptoms, rather than relying solely on “resorption” as the efficacy criterion.

Although this study provides evidence regarding outcomes associated with the inflammation-preserving protocol through a 6-year follow-up, several limitations warrant consideration. The most significant limitation of this study is its single-arm, single-center design without a concurrent control group receiving traditional NSAID-based conservative treatment. Consequently, we cannot differentiate the effects attributable to the inflammation-preserving protocol from the natural history of spontaneous resorption, which is known to occur in 60%−90% of patients with extruded/migrated LDH. The observed differences between rim enhancement positive and negative groups demonstrate prognostic stratification rather than causal treatment efficacy. Therefore, our findings should be interpreted as exploratory and hypothesis-generating, providing a strong rationale for future randomized controlled trials to establish causality. A second major limitation, particularly relevant for an immunology context, is the reliance on MRI rim enhancement as the sole surrogate for inflammation. The lack of correlative systemic (e.g., CRP, IL-6, TNF-α) or local biochemical markers hinders precise definition of the “moderate inflammation” state and validation of the imaging findings against actual immune activity. Consequently, our inferences regarding the underlying inflammatory state are indirect and should be considered exploratory. Other considerations include: the potential confounding effect of unrecorded long-term analgesic use on subjective outcomes, despite no acute-phase inter-group difference; the financial and logistical burden of serial contrast-enhanced MRI; and the inability to differentiate symptom contributions from residual mechanical compression vs. ongoing nerve root edema.

From a broader clinical and socio-economic perspective, while this study did not specifically assess endpoints such as time to return to work, the sustained stability in functional improvement (JOA, ODI) and neurological recovery over 6 years suggests a rehabilitation trajectory conducive to early reintegration into daily activities and occupational roles. Future studies should incorporate patient-reported outcome measures (PROMs) and objective assessments of functional capacity to comprehensively evaluate the social and economic impact of the inflammation-preserving protocol.

To address these limitations, future research could focus on three directions: First, conducting multicenter, large-sample randomized controlled trials with a traditional NSAIDs treatment group to directly compare the efficacy differences between the two protocols. Second, integrating inflammation-related biochemical markers with imaging techniques to establish an integrated “imaging-biochemical” assessment system for precisely defining the range of “moderate inflammation.” Such an approach would validate the imaging findings against actual immune activity and allow for more precise patient stratification. Third, exploring targeted inflammation modulation strategies, such as local application of low-dose anti-inflammatory drugs (to suppress excessive inflammation while preserving beneficial inflammation) or combining TCM syndrome differentiation and treatment (e.g., the Yiqi Huoxue recipe used in this study), to promote resorption while mitigating inflammation-related risks, ultimately advancing the conservative management of extruded/migrated LDH toward precision and individualization.

In conclusion, this study provides long-term follow-up data demonstrating that patients with extruded/migrated LDH managed with an inflammation-preserving protocol achieve favorable outcomes, particularly those with positive rim enhancement. The rim enhancement sign on contrast-enhanced MRI serves as a useful non-invasive biomarker for identifying patients likely to experience significant resorption and symptomatic improvement. Given the absence of a control group, future randomized controlled trials comparing this protocol with conventional NSAID-based treatment are warranted to further validate the inflammation-preserving protocol.

## Data Availability

The original contributions presented in the study are included in the article/supplementary material, further inquiries can be directed to the corresponding authors.
